# Resveratrol Enhances Apoptotic and Oxidant Effects of Paclitaxel through TRPM2 Channel Activation in DBTRG Glioblastoma Cells

**DOI:** 10.1155/2019/4619865

**Published:** 2019-03-07

**Authors:** Yasin Öztürk, Caner Günaydın, Fatma Yalçın, Mustafa Nazıroğlu, Nady Braidy

**Affiliations:** ^1^Department of Pharmacology and Toxicology, Faculty of Veterinary Medicine, Bingöl University, Bingöl, Turkey; ^2^Department of Pharmacology, Faculty of Medicine, Ondokuz Mayıs University, Samsun, Turkey; ^3^Department of Biophysics, Faculty of Medicine, Osmangazi University, Eskişehir, Turkey; ^4^Neuroscience Research Center, Suleyman Demirel University, Isparta, Turkey; ^5^Drug Discovery and Development Research Group in Neuroscience, BSN Health, Analysis and Innovation, Goller Bolgesi Teknokenti, Isparta, Turkey; ^6^Centre for Healthy Brain Ageing, School of Psychiatry, University of New South Wales, Sydney, Australia

## Abstract

Numerous studies have reported a strong association between increased production of reactive oxygen species (ROS) and the pathobiology of several diseases, and cancer in particular. Therefore, manipulation of cellular oxidative stress levels represents an important therapeutic target. Recently, resveratrol (RESV), a naturally occurring phytochemical, has been shown to sensitize several cell lines to the anticancer effects of other chemotherapeutic agents, including paclitaxel (PAX). However, the molecular mechanisms of action of RESV through oxidative sensitive TRPM2 channel activation remain unclear. The aim of this study was to evaluate the effect of combination therapy of RESV and PAX on activation of TRPM2 in DBTRG glioblastoma cells. DBTRG cells were divided into four treatment groups: control, RESV (50 *μ*M), PAX (50 *μ*M), and PAX + RESV for 24 hours. Our data shows that markers for apoptosis, mitochondrial membrane depolarization and mitochondrial function, intracellular steady-state ROS levels, caspase 3 activity, TRPM2 current density, and Ca^2+^ florescence intensity were significantly increased in DBTRG cells following treatment with PAX and RESV, respectively, although cell viability was also decreased by these treatments. These biochemical markers were further increased to favor the anticancer effects of PAX in DBTRG cells in combination with RESV. The PAX and RESV-mediated increase in current density and Ca^2+^ florescence intensity was decreased with a TRPM2 blocker. This suggests that for this combination therapy to have a substantial effect on apoptosis and cell viability, the TRPM2 channel must be stimulated.

## 1. Introduction

Gliomas are primary solid tumors of the brain and are induced in the brain by glial cell activation. Glioblastoma multiforme (GBM) is the most aggressive, fast-growing glioma that is formed from glial cells which support the viability of neuronal cells within the central nervous system (CNS) [1]. GBM is defined as a grade IV astrocytoma. Even with optimal therapy, the median duration of survival is between 12 and 15 months, and less than 5% of all patients survive for more than 5 years. Without treatment, survival is often limited to less than 3 months [2]. Since surgery, radiotherapy, and chemotherapy are unlikely to lead to a significant increase in remission of GBM tumors, there is a strong need for the use of new innovative treatments with less adverse effects, when first-line therapy was unsuccessful.

Paclitaxel (PAX) is an effective and widely used anticancer drug isolated from the bark and trunk of Pacific yew [3]. PAX is widely used in clinical practice for the treatment of several types of cancer including GBM [4], lung [5], ovary [6], and breast [7]. Mechanistically, PAX is thought to stimulate and enhance tubulin polymerization, leading to G2/M or G1 cell cycle arrest and apoptosis [8]. PAX has also been shown to induce several factors including reactive oxygen species (ROS) production and apoptosis level in cancer cells [9]. Resistance to PAX-induced apoptosis and oxidative stress production has been previously reported in glioblastoma cells [10]. Therefore, the high mortality rate of glioblastoma cancer can be partly attributed to the unknown molecular pathways of PAX resistance. Recently, it was demonstrated that resveratrol (RESV) could sensitize several cancer cell lines to the anticancer effects of other cancer drugs, including paclitaxel [11–15]. It has been suggested by our group and others that since RESV and PAX have differential effects on apoptosis and cell cycle regulation, their combination may have synergistic anticancer activity.

RESV (trans-3,4′,5-trihydroxystilbene) is a naturally occurring phytochemical that is abundant in grapes, peanuts, and red wine. Accumulating evidence suggests that RESV can mediate several health benefits including lifespan extension, anti-inflammation, and protection against cardiovascular and neurodegenerative diseases [16, 17]. In addition to the antioxidant role of RESV, prooxidant and non-antioxidant effects of RESV have been previously reported and are dependent on the cellular model used, treatment conditions, and dosage/formulation/time of delivery [18–25]. In addition, the non-antioxidant role of RESV was also reported in patients with nonalcoholic fatty liver disease [26, 27]. Evidence in support of the chemopreventative effect of RESV in a number of cellular and animal models has been convincing. For instance, one study observed excessive ROS production and apoptosis in cancer cells exposed to RESV [28–30]. RESV also induced oxidative DNA damage in the form of DNA strand breaks in the presence of cupric cations [31]. RESV has also been shown to inhibit cancer cell growth when used alone at high concentrations (>50 *μ*M) or in combination with other anticancer drugs [32–42]. RESV promoted ROS production to induce cellular resistance through the p53-CXCR2 pathway in human osteosarcoma and pulmonary cancer cell lines [43]. Since resistance to PAX in the treatment of GBM cells is a major obstacle, combination with natural phytochemicals such as RESV may improve treatment outcomes. However, the molecular mechanisms underlying RESV and PAX-mediated drug resistance remain to be clarified.

Calcium ion (Ca^2+^) is a major intracellular second messenger signaling cation that regulates many physiological functions including cell migration and apoptosis [44]. Transient receptor potential (TRP) melastatin 2 (TRPM2) is a subfamily member of the TRP superfamily with residual ADP-ribose (ADPR) hydrolase activity [45]. In addition to ADPR, TRPM2 is activated in primary and cancer cells by reactive oxygen species [46–52]. In different cancer cells, an increase in [Ca^2+^]i through activation of TRPM2 channels increased the rate of cell migration, presumably tumor invasion [53]. Hence, TRPM2 channels serve as targets of therapeutic agents to limit tumor growth and tumor-induced Ca^2+^ entry in cancer treatments [54]. Moreover, alpha lipoic acid has been shown to attenuate hypoxia-induced apoptosis, inflammation, and mitochondrial oxidative stress via inhibition of the TRPA1 channel in a human glioblastoma cell line [55].

In this study, we report that PAX and RESV increased intracellular steady-state ROS levels and mitochondrial dysfunction culminating in a decline in cell viability and increased cell death via an apoptotic mechanism. To our knowledge, the cytotoxic effects of RESV on PAX-induced oxidative stress and TRPM2 channel activation in DBTRG cells have not been previously investigated. The aim of this study was to evaluate if RESV treatment could increase PAX-induced oxidative damage in DBTRG glioblastoma cells *in vitro*. We show that glioblastoma cell apoptosis and mitochondrial oxidative stress in these cells can be modulated by treatment with PAX and RESV. Increased TRPM2 channel activation in these cells may represent an important mechanism for modulating GBM cell death.

## 2. Materials and Methods

### 2.1. Cell Culture

The DBTRG cell line was purchased from Şap Institute, Ministry of Agriculture and Forestry (Ankara, Turkey). These cells were cultured in DMEM medium (Invitrogen, Istanbul, Turkey) added with 10% fetal bovine serum (Gibco, Istanbul, Turkey), 1% antibiotic combination (penicillin-streptomycin) (Sigma-Aldrich, Istanbul, Turkey), and 100 *μ*g/ml sodium pyruvate (Sigma-Aldrich, Istanbul, Turkey) at 37°C in a 5% CO_2_ incubator (HF90, Heal Force Bio-meditech Holdings Limited, Shanghai, China). Before starting the treatments, the cells were examined within 24 hours after plating onto the coverslips. The cells were counted by using an automatic cell counter (CASY Model TT, Roche, Germany). Cells were seeded in 3-6 flasks at a density of 1 × 10^6^ cells per flask (filter cap, sterile, 250 ml, 75 cm^**2**^). All cells were cultured at 37°C.

### 2.2. Groups

ADP, cumene hydroperoxide (CPx), N-(p-amylcinnamoyl)anthranilic acid (ACA), PAX, and RESV were purchased from Santa Cruz Inc. (Istanbul, Turkey).

The DBTRG cells were divided into four groups as follows: (1) control group which were not incubated with ADPR, cumene hydroperoxide (CPx), ACA, PAX, and RESV but were kept in a flask containing the same cell culture medium and conditions for 24 hours; (2) PAX group in which cells were kept 24 hours in the same culture conditions without treatment and then incubated with PAX (50 *μ*M) for 24 hours [56]; (3) RESV group in which cells were incubated for 24 hours in the same culture condition without treatment and then preincubated with RESV (50 *μ*M) for 24 hours as described in a previous study [57]; and (4) PAX + RESV group in which cells were incubated with PAX (50 *μ*M) and RESV (50 *μ*M) for 24 hours.

### 2.3. Electrophysiology

Patch-clamp records as whole-cell voltage clamp configuration were obtained from the DBTRG cells at room temperature (22 ± 2°C) (EPC 10 patch-clamp set, HEKA, Lamprecht, Germany). Details of standard intracellular and extracellular buffers were given in previous studies [46, 58]. Na^+^-free solutions were prepared by replacement of 150 mM N-methyl-D-glucamine (NMDG^+^) instead of NaCl. It is well known that the TRPM2 channel is activated by the presence of high intracellular Ca^2+^. Therefore, high intracellular Ca^2+^ concentration (1 *μ*M instead of 100 nM) was used in the patch-clamp experiments. The holding potential of the patch-clamp analyses in the DBTRG was kept as -60 mV. Voltage clamp technique was used in the analyses, and current-voltage (I-V) relationships were obtained from voltage ramps from -150 to +150 mV applied over 200 milliseconds.

In the experiments, TRPM2 was intracellularly gated by ADPR (1 mM) (in a patch pipette), and the channels were extracellularly blocked by ACA (25 *μ*M). The maximal current amplitudes (pA) in a DBTRG were divided by the cell capacitance (pF), a measure of the cell surface. Values of current density were expressed as pA/pF in the patch-clamp experiments.

### 2.4. Intracellular Ca^2+^ Fluorescence Intensity Measurement through TRPM2 Activation

Intracellular changes in the Ca^2+^ florescence intensity concentration in the DBTRG cell were monitored using 1 *μ*M florescent dye (Fluo3, Calbiochem, Darmstadt, Germany). The cells were treated with the TRPM2 antagonist ACA (25 *μ*M) to inhibit Ca^2+^ entry before stimulation with the TRPM2 channel agonist CPx (1 mM). The cells were analyzed by a laser confocal microscope (LSM 800, Zeiss, Ankara, Turkey) fitted with a 40x oil objective. The results of Fluo3 in 15 *μ*m^2^ of cytosol were indicated as the mean fluorescence intensity as arbitrary unit per cell.

### 2.5. MTT Assay of Cell Viability

Cell viability was assayed using the 3-(4,5-dimethylthiazol-2yl)-2,5-diphenyltetrazolium bromide colorimetric (MTT) colorimetric assay [59]. Briefly, the culture medium was removed from the cells by washing with 1x PBS, and 100 *μ*l DMEM medium containing 0.5 mg/ml MTT was added. The cells were then incubated for another 4 h. Finally, absorbance values were detected at 490 nm by a microplate reader (Infinite Pro 200; Tecan Austria GmbH, Groedig, Austria) and the ratio of suppression by the DTX and RESV treatments on cell viability was calculated. We performed a total of 6 experiments (*n* = 6) for the cell viability assay. The data are presented as fold increase normalized to control.

### 2.6. Extracellular LDH Activity as a Measurement for Cytotoxicity

The release of lactate dehydrogenase (LDH) into the culture supernatant correlates with the amount of cell death and membrane damage, providing an accurate measure of cellular toxicity. LDH activity was assayed using a standard spectrophotometric technique described by Koh and Choi [60].

### 2.7. Assay of Apoptosis Level and Caspase 3 and 9 Activities

For determining spectrophotometric (UV-1800, Shimadzu, Kyoto, Japan) analysis apoptosis, we used the Cell APOPercentage Apoptosis kit purchased from Biocolor Ltd. (Northern Ireland) [59]. Briefly, the assay used a dye that is selectively imported by cells that are undergoing apoptosis. Necrotic cells cannot retain the dye and therefore are not stained. The dye that accumulates in 30 minutes within labeled cells is released into solution and the concentration of released intracellular dye measured at 550 nm (or blue-green filter) by a microplate colorimeter (Infinite Pro 200).

The determination of caspase 3 and 9 activities was based on a method previously reported with minor modifications [61]. Caspase 3 (N-acetyl-Asp-Glu-Val-Asp-7-amido-4-methylcoumarin) and 9 (N-acetyl-Leu-Glu- His-Asp-7-amino-4-methylcoumarin) substrates were purchased from Bachem (Bubendorf, Switzerland), and cleavage of the substrates was measured with the microplate reader (Infinite Pro 200) (excitation = 360 nm and emission = 460 nm). The data were calculated as fluorescence units/mg protein and presented as fold increase over the pretreatment level. We performed a total of 6 experiments (*n* = 6) for the caspase and apoptosis analyses.

### 2.8. Measurement of Mitochondrial Membrane Potential (ΔΨm)

5,5′,6,6′-Tetrachloro-1,1′,3,3′-tetraethylbenzimidazolylcarbocyanine iodide (JC1) accumulates in mitochondria according to the ΔΨm level and is present either as monomer or as reversible J-aggregate. The JC1 monomer predominating in depolarized mitochondria emits green fluorescence at 530 nm, whereas the oligomer (J-aggregate) forming in mitochondria with negative potentials emits red fluorescence at 590 nm [62]. The mitochondrial membrane potential was assayed using the fluorescent probe JC1 according to the manufacturer's instruction (Thermo Fisher, Istanbul, Turkey). Briefly, cells were cultured in 96-well plates. After being treated with PAX and RESV, the cells were cultured in the cell culture medium containing the JC1 probe at 37°C for 25 min, then centrifuged for 5 min at 300 *g* at 4°C. The cells were incubated with the JC1 staining buffer for two minutes. Finally, the green fluorescent intensities (JC1 monomer, excitation = 485 nm, emission = 530 nm) and red signal (JC1 aggregate, excitation = 540 nm, emission = 590 nm) were analyzed, respectively, using the microplate reader (Infinite Pro 200). The ratio of red to green fluorescence intensity is a measure of the mitochondrial membrane potential. The data are presented as fold increase normalized to control.

### 2.9. XF24 Microplate-Based Respirometry

To determine the effect of PAX and RESV on oxygen consumption rates (OCRs; as indicator of mitochondrial respiration) in DBTRG cells, the Seahorse XF24 extracellular flux analyzer (Seahorse Bioscience, North Billerica, MA, USA) was employed as previously described. Briefly, culture plates were incubated in a CO_2_-free incubator at 37°C for 1 hr to equilibrate for temperature and pH. The microplate was then loaded into the XF24 and further incubated for 15 min by a 3 min mix and 2 min wait cycles before commencement of the assay. The XF assay was performed as previously described [63]. After determination of the basal respiration in the cell culture, oligomycin (2 *μ*M), carbonyl cyanide-p-trifluoromethoxyphenylhydrazone (FCCP, 500 nM), and antimycin (3 *μ*M) were sequentially added and the oxygen consumption rates (OCRs) for each culture well were quantified for 2 minutes. This allowed us to determine the basal control ratio (BCR, i.e., basal/maximum respiration) and the uncoupling ratio (UCR, i.e., mitochondrial functional integrity) [64]. Essentially, the BCR is a measurement of how close the basal level of respiration is to the maximum level of respiration (i.e., basal/maximum).

### 2.10. Detection of Intracellular Steady-State Reactive Oxygen Species (ROS) Using the DCFH-DA and *o*- and *m*-Tyrosine Assays

2′,7′-Dichlorofluorescin diacetate (DCFH-DA) is a cell-permeable ROS-specific nonfluorescent probe. The DCFH-DA is hydrolysed intracellularly, and later it is oxidized by ROS into 2′-7′dichlorofluorescein. Intracellular steady-state ROS levels were detected using the DCFH-DA assay (Sigma-Aldrich, Istanbul, Turkey). The cells were treated PAX and RESV for 24 h. After washing the cells, they were incubated with DCFH-DA (10 *μ*M) for 25 min at 37°C in the dark. The cells were washed again and analyzed (excitation = 485 nm, emission = 535 nm) using the microplate reader (Infinite Pro 200). The data were presented as fold increase normalized to control.

We also measured *o-* and *m-*tyrosine as previously described [65]. Briefly, phenylalanine (313 pmol and 50 nmol) and *o-* and *m-*tyrosine (313 fmol to 50 pmol) were used as calibration standards. Labeled [^13^C_6_]-phenylalanine was used as an internal standard. Each standard and sample (1 *μ*l) was injected into the GC/MS using a pressure pulsed splitless loading 6890 N gas chromatograph interfaced to a 5973 mass selective detector (Agilent technologies), with methane as the ECNI reagent gas. *o-* and *m-*Tyrosine levels were expressed relative to phenylalanine levels.

### 2.11. Bradford Protein Assay for the Quantification of Total Protein

Enzyme assays were adjusted for variations in cell number using the Bradford protein assay described by Bradford [66].

### 2.12. Statistical Analyses

The results were presented as mean ± standard deviation (SD). Statistical differences between the mean values for individual groups were assessed using Fisher's least significant difference (LSD) test in the SPSS program 18.0 (IBM, New York, NY, USA). Statistical significances were determined using a nonparametric test (Mann-Whitney *U* test), and *p* ≤ 0.05 was accepted as significant.

## 3. Results

### 3.1. Effects of PAX and RESV on TRPM2 Currents in DBTRG Cells

**Figure 1:**
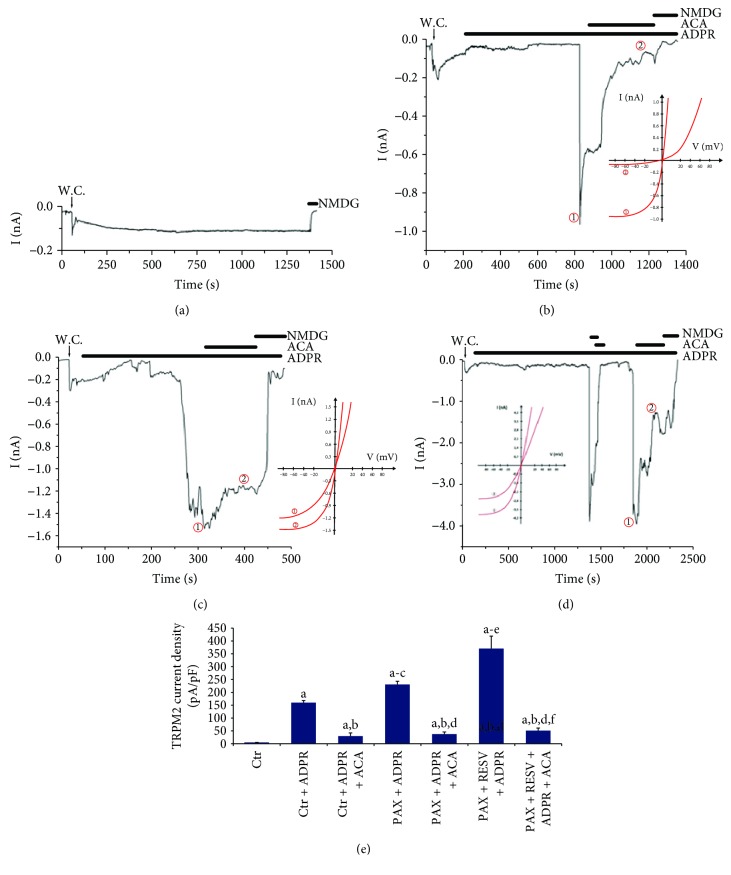
Effect of resveratrol (RESV and 50 *μ*M for 24 hours) and paclitaxel (PAX and 50 *μ*M for 24 hours) incubations on the TRPM2 current densities (pA/pF) in the DBTRG cells (mean ± SD and *n* = 3). The TRPM2 currents in the cells were induced by intracellular ADPR (1 mM in patch pipette), but they were blocked by extracellular ACA (25 *μ*M) in the patch-chamber. W.C.: whole cell. (a) Control (Ctr): original recordings from Ctr cell (without ADPR stimulation). (b) Ctr + ADPR group (with ADPR stimulation but not PAX and RESV treatments). (c) Ctr + ADPR + ACA group (with ADPR stimulation and PAX treatment but not RESV treatment). (d) PAX + ADPR group (with ADPR stimulation and PAX plus RESV treatments). (e) Current densities. (^a^*p* ≤ 0.001 versus control. ^b^*p* ≤ 0.001 versus control + ADPR group. ^c^*p* ≤ 0.001 versus control + ADPR + ACA group. ^d^*p* ≤ 0.001 versus PAX + ADPR group. ^e^*p* ≤ 0.001 versus PAX + ADPR + ACA group. ^f^*p* ≤ 0.001 versus PAX + RESV + ADPR group).

Results of current density reported as pA/pF in the patch-clamp records are shown in Figure 1. There were no significant currents in the absence of the TRPM2 agonists and antagonists (ADPR and ACA) (Figure 1(a)). The TRPM2 channel in the patch-clamp experiments was gated in the cells by intracellular ADPR, although they were reversibly blocked by ACA and NMDG^+^ (replacement of Na^+^). As expected, the current densities in the DBTRG cells were increased to about 150 pA/pF in the Ctr + ADPR group compared with the Ctr group (5 pA/pF) alone (*p* ≤ 0.001). However, the current density of TRPM2 was 6-fold (*p* ≤ 0.001) lower in the Ctr + ADPR + ACA group compared to the Ctr + ADPR group (Figure 1(b)). We observed a 10-fold increase in the current densities in DBTRG cells in the PAX + ADPR group compared to the Ctrl + ADPR + ACA group (Figure 1(c)). The TRPM2 currents increased by 1.6-fold in the PAX + RESV + ADPR group as compared to the PAX + ADPR group (Figures 1(d) and 1(e)). These results clearly indicate that PAX induces Ca^2+^ influx through TRPM2 activation. In addition, PAX-induced TRPM2 currents through ROS production were further increased by treatment with RESV.

### 3.2. Effects of PAX and RESV on Florescence Intensity of Ca^2+^ through TRPM2 Activation in DBTRG Cells

**Figure 2:**
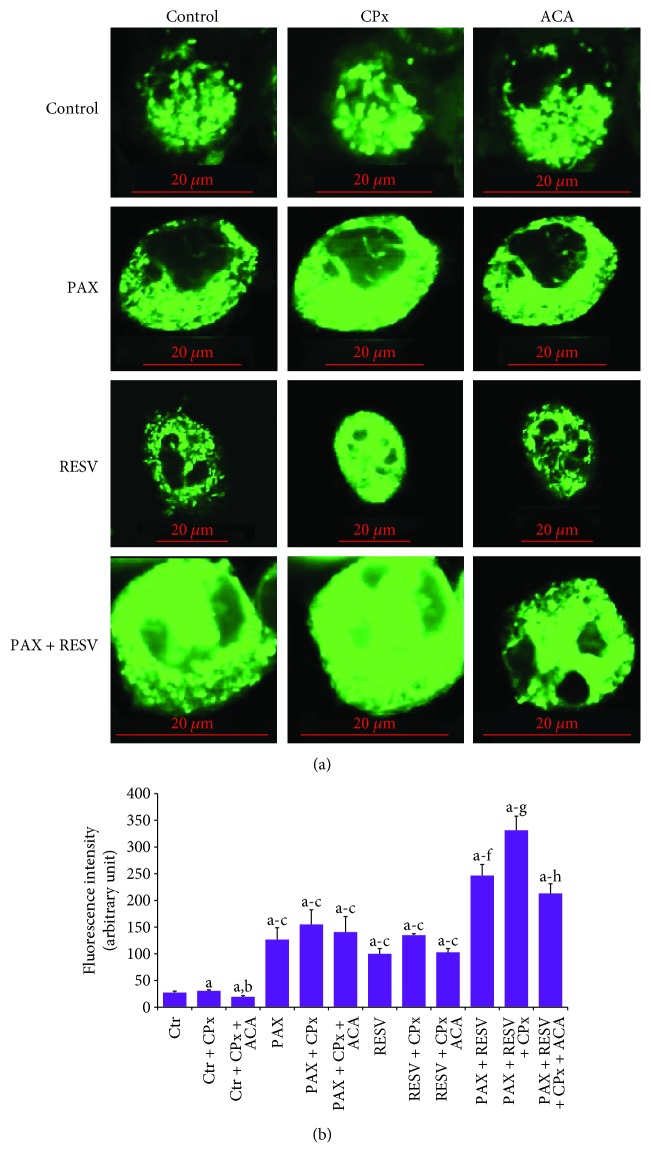
Effect of resveratrol (RESV and 50 *μ*M for 24 hours) and paclitaxel (PAX and 50 *μ*M for 24 hours) incubations on the Ca^2+^ florescence intensity through TRPM2 activation in the DBTRG cells. The cells were stained with Fluo3 calcium dye, and the mean ± SD of fluorescence in 15 mm^2^ of cell as arbitrary unit are presented; *n* = 10 independent experiments. The cells were extracellularly stimulated by cumene hydroperoxide (CPx and 1 mM), but they were extracellularly inhibited by ACA (0.025 mM). The samples were analyzed by a laser confocal microscope fitted with a 40x oil objective. (^a^*p* ≤ 0.001 versus control (Ctr) group. ^b^*p* ≤ 0.001 versus Ctr + CPx. ^c^*p* ≤ 0.001 versus Ctr + CPx + ACA group. ^d^*p* ≤ 0.001 versus RESV + PAX group. ^e^*p* ≤ 0.001 versus RESV + CPx and PAX + CPx groups. ^f^*p* ≤ 0.001 versus RESV + CPx + ACA and PAX + CPx + ACA groups. ^g^*p* ≤ 0.001 versus PAX + RESV group. ^h^*p* ≤ 0.001 versus PAX + RESV + CPx group).

Results of Ca^2+^ florescence intensity in the laser confocal microscopy analyses of four groups are indicated in Figure 2. The Ca^2+^ florescence intensity in the DBTRG cell was 1.3-fold higher in the Ctr + CPx group compared to the Ctr group alone (*p* ≤ 0.001); however, the Ca^2+^ florescence intensity of TRPM2 was 2-fold (*p* ≤ 0.001) lower in the Ctr + CPx + ACA group compared to the Ctr + CPx group. In addition, the Ca^2+^ florescence intensities were increased in the PAX (4.2- and 3.2-fold), RESV (3.3- and 2.5-fold), PAX + CPx (5- and 3.8-fold), and RESV + CPx (4.3- and 3.3-fold) compared to Ctr and Ctr + CPx groups, respectively (*p* ≤ 0.001). The Ca^2+^ florescence intensity in the DBTRG cell was further increased in the PAX + RESV group compared to the PAX (2-fold) or RESV (2.5-fold) group (*p* ≤ 0.001); however, the Ca^2+^ florescence intensity of TRPM2 was significantly (*p* ≤ 0.001) reduced in the ACA treatment groups (PAX + CPx + ACA, RESV + CPx + ACA, and PAX + RESV + CPx + ACA) compared to the CPx (PAX + CPx, RESV + CPx, and PAX + RESV + CPx) groups. PAX and RESV-mediated Ca^2+^ influx through TRPM2 activation was further confirmed by the results of Ca^2+^ florescence intensity.

### 3.3. Apoptosis, Caspase, and Cell Viability (MTT) Results

**Figure 3:**
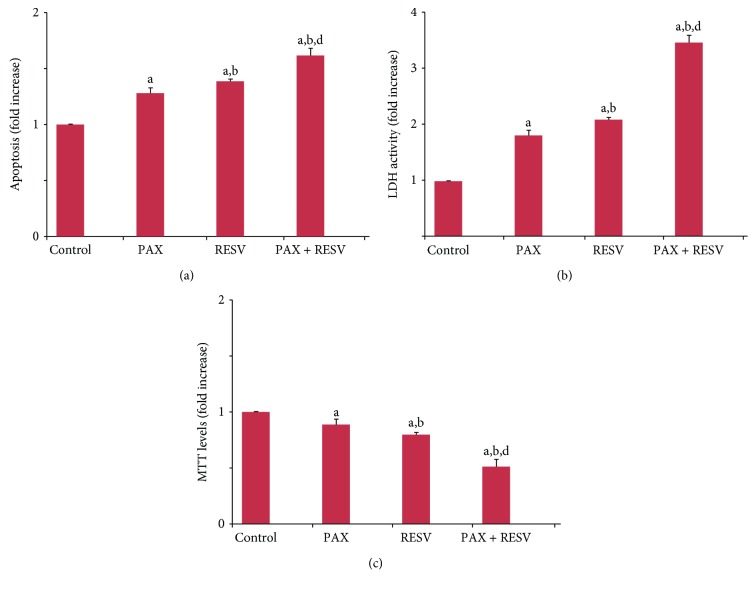
Effect of resveratrol (RESV and 50 *μ*M for 24 hours) and paclitaxel (PAX and 50 *μ*M for 24 hours) treatments on (a) apoptosis, (b) LDH activity, and (c) cell viability (MTT) levels in the DBTRG cells (mean ± SD and *n* = 3). (^a^*p* ≤ 0.001 versus control. ^b^*p* ≤ 0.001 and ^c^*p* ≤ 0.05 versus PAX group. ^d^*p* ≤ 0.001 versus RESV group).

Compared with control, PAX and RESV treatment increased the levels of apoptosis (1.3- and 1.5-fold) (Figure 3(a)) and extracellular LDH activity (1.8- and 2.1-fold) (*p* ≤ 0.001) (Figure 3(b)), although MTT levels were decreased by the treatments (0.8- and 0.7-fold) (Figure 3(c)). More importantly, we found a significant additive effect on apoptosis (1.3- and 1.2-fold) and MTT levels (0.6- and 0.7-fold) and LDH activities (1.9- and 1.6-fold) in the PAX + RESV combination group compared to PAX and RESV treatment alone (*p* ≤ 0.001). Our data suggests that PAX-induced apoptosis can be further increased by addition of RESV.

### 3.4. Caspase 3 and 9 Activities

**Figure 4:**
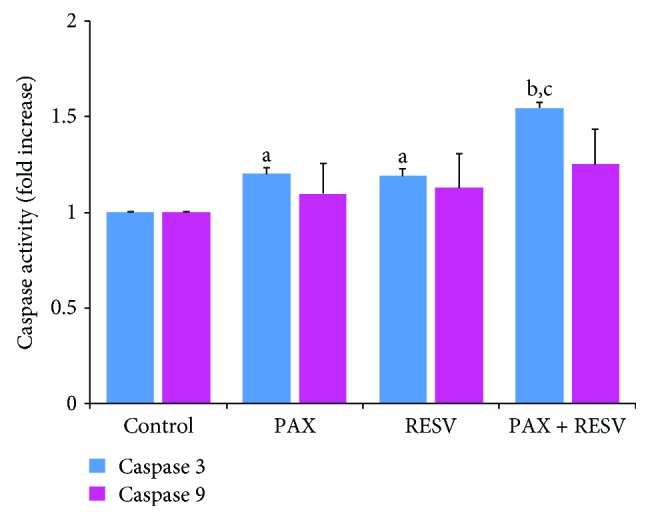
Effect of resveratrol (RESV and 50 *μ*M for 24 hours) and paclitaxel (PAX and 50 *μ*M for 24 hours) treatments on the caspase 3 and 9 activities in the DBTRG cells (mean ± SD and *n* = 3). (^a^*p* ≤ 0.05 and ^b^*p* ≤ 0.001 versus control. ^c^*p* ≤ 0.001 versus PAX and RESV groups).

It is well known that mitochondria-dependent intrinsic apoptosis is mediated by excessive Ca^2+^ influx-induced mitochondrial membrane depolarization, leading to activation of caspase 9 and subsequent activation of caspase 3 [46, 58]. After observing increases in Ca^2+^ influx and apoptosis, we decided to measure the activities of intrinsic apoptosis caspase markers, caspase 3 and 9 (Figure 4). Our data shows that caspase 3 but not caspase 9 activity was markedly (*p* ≤ 0.05) increased by 1.25-fold in the PAX and RESV groups compared to the control group. Caspase 3 activity was further increased by 1.28-fold in the PAX + RESV group compared to PAX and RESV groups alone (*p* ≤ 0.001).

### 3.5. Mitochondrial Depolarization (JC1) and Intracellular Steady-State ROS Levels

**Figure 5:**
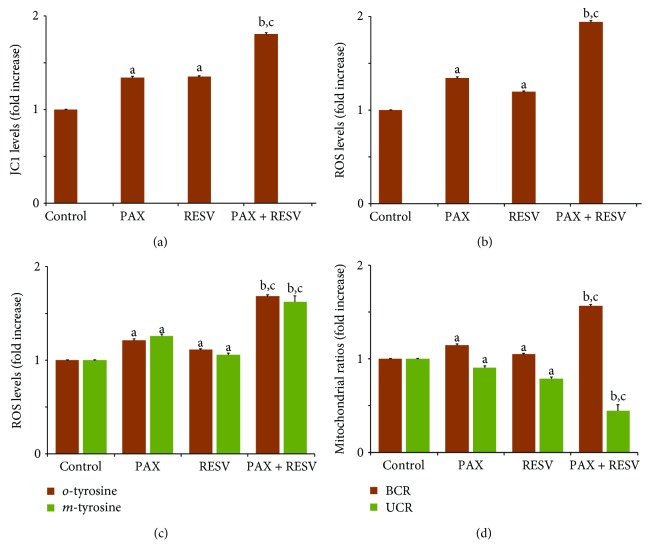
Effect of resveratrol (RESV and 50 *μ*M for 24 hours) and paclitaxel (PAX and 50 *μ*M for 24 hours) treatments on (a) mitochondrial membrane depolarization (JC1), (b) intracellular DCFH-DA ROS production, (c) *o*- and *m*-tyrosine levels, and (d) BCR and UCR ratios in the DBTRG cells (mean ± SD and *n* = 3). (^a^*p* ≤ 0.05 and ^b^*p* ≤ 0.001 versus control. ^c^*p* ≤ 0.001 versus PAX and RESV groups).

PAX and RESV increased overloading Ca^2+^ entry through TRPM2 channel activation to promote cancer cell death. It is well known that the overloading Ca^2+^ entry induces mitochondrial dysfunction and intracellular ROS production [67]. Excessive ROS production is known to increase cellular sensitivity in cancer treatment [68]. To investigate whether RESV enhances the effects of PAX on mitochondrial ROS level, we measured the JC1 level and intracellular steady-state ROS levels using a well-established spectrophotometric assay (Infinite Pro 200). We found that the levels of JC1 (Figure 5(a)), DCFH-DA ROS (Figure 5(b)), and *o*- and *m*-tyrosine (Figure 5(c)) were significantly (*p* ≤ 0.05) elevated by about 1.5-fold following treatment with PAX and RESV in DBTRG cells. In addition, the levels of JC1 and ROS were further increased by combination treatment of PAX and RESV (*p* ≤ 0.001). Similarly, we also observed a significant increase in basal control ratio (BCR) and a decline in uncoupling ratio (UCR) in DBTRG cells after a 24 hr incubation with RESV (1.3- and 0.7-fold) and PAX (1.3- and 0.8-fold), and more so in the RESV + PAX group (1.7- and 0.5-fold) using the Seahorse XF24 (Seahorse Bioscience) (Figure 5(d)), consistent with enhanced mitochondrial dysfunction.

## 4. Discussion

Resistance to chemotherapy is an emerging problem for the treatment of GBMs, and identification of alternative adjuvant antitumoral therapeutic agents is necessary for the treatment of cancers [69–73]. In addition to antioxidant roles in normal cells, the prooxidant role of RESV has been previously reported in cancer cells [29]. RESV selectively triggers death in cancer cells and has been previously used for stimulation of apoptosis, mitochondrial oxidative stress, and Ca^2+^ entry in several cancer cell lines [25, 43, 67]. We provide evidence that PAX induced mitochondrial ROS and apoptosis in the DBTRG cells when the TRPM2 channel is activated. We observed that mitochondrial ROS could also activate TRPM2, resulting in increased apoptosis and caspase 3 activation and reduced cell viability. The prooxidant and apoptotic roles of PAX were further enhanced by RESV treatment. This would contribute to cancer cell death by increasing the influx of Ca^2+^ into the cell through TRPM2 channel activation.

TRPM2 channel activation is very sensitive to mitochondrial oxidative stress [44, 45, 59]. PAX induces mitochondrial ROS production which is cytotoxic to some cancer cells. RESV has antioxidant roles in the treatment of various metabolic and cardiovascular disorders. However, recent data indicates that it exhibits prooxidant roles that are likely to be beneficial for the treatment of cancer cells [13, 18, 68]. In the current study, we observed increased levels of TRPM2 current density and Ca^2+^ florescent intensity in the DBTRG cells. More specifically, we observed activation of the TRPM2 channel by PAX and RESV-induced mitochondrial ROS production. To our knowledge, there is no report on RESV, PAX, and TRPM2 channel in DBTRG glioblastoma cells. Similarly, it was previously reported that TRPM2 knockdown significantly enhances gastric cancer cell sensitivity to PAX which validates its therapeutic potential as an anticancer target [54]. These results are consistent with reports on the benefits of targeting TRPM2 in the treatment of neuroblastoma [49, 74–76] and breast cancer cell lines [77]. Contrary to the current results, MPP^+^ treatment increased the level of ROS-mediated TRPM2 activation, and treatment with resveratrol (RSV) attenuated TRPM2-mediated Ca^2+^ influx, reduced intracellular Ca^2+^ overload, and promoted cell survival [78]. These findings suggest that the effects of RESV on TRPM2 may be cell-specific.

The role of the mitochondria in PAX-induced drug resistance is widely related to their major role as a producer of ROS-mediated molecular injury. Increased mitochondrial membrane depolarization, impaired mitochondrial function, and increased ROS production in PAX-induced cell death have been well documented. However, reports on RESV-induced mitochondrial ROS production are conflicting. RESV has been reported to increase mitochondrial membrane dysfunction to induce cell death by increasing the ROS level in cancer cells [79]. A central regulator of mitochondrial biogenesis is a peroxisome proliferator-activated receptor gamma coactivator-1 (PGC-*α*), and RESV treatment increased mitochondrial membrane depolarization but decreased mitochondrial gene transcript activity and PGC-*α* expression in lung, breast epithelial, and breast cancer cell lines [68]. Mitochondrial oxidative stress in cancer cells can be induced by PAX [80–82] and RESV, through alterations in mitochondrial membrane depolarization and ATP production, which leads to increased oxidative phosphorylation through the electron transport chain and hence the formation of JC1 and reduced mitochondrial respiration. Thus, activation of apoptosis by PAX and RESV is likely to increase toxic protein aggregates inhibiting cell survival. In the current study, we observed increased levels of apoptosis, mitochondrial membrane depolarization, and ROS values in DBTRG cells exposed to PAX treatment and the effect was further increased in cells cotreated with RESV. Accordingly, the apoptotic and prooxidant roles of RESV have been previously reported in human multiple myeloma cells [83].

Two main pathways of apoptosis are extrinsic and intrinsic apoptosis. Several proteins act in extrinsic apoptosis; for instance, caspase 8 shears and activates downstream apoptosis-inducing proteins including caspase 3 [84]. Caspase 3 acts through both extrinsic and intrinsic apoptosis pathways [85]. In the current study, caspase 3 but not caspase 9 activity was increased by PAX treatment. The effect of PAX treatment on caspase 3 activation was further increased by RESV treatment. Hence, this combination enhanced apoptotic effects in DBTRG cells. Similarly, another study showed that RESV increased the anticancer effects of PAX through upregulation of apoptosis and caspase 3 in non-small cell lung cancer [86] and HepG2 cancer cell lines [12, 87, 88]. Our data shows that PAX and RESV treatment enhances apoptosis in a caspase-9-independent manner. Impaired caspase-9 activation and resistance to anticancer drugs have been previously shown to be independent of the expression of p53, Bcl-2 family proteins, Fas receptor, and Fas ligand and is likely to induce a higher cellular threshold for PAX- and RESV-mediated cell death in DBTRG cells [89]. However, treatment with higher doses may affect this threshold. For example, one study showed that higher doses of cisplatin doses could overcome this threshold, via an alternate, caspase-9-independent apoptotic pathway [89]. These findings are in support for clinical use of high-dose chemotherapy in patients with chemorefractory tumors [89]. However, further understanding of the exact molecular mechanism of action of PAX and RESV on caspase-9 activation is required to overcome chemoresistance in GBMs.

In summary, we observed that mitochondrial ROS and apoptosis produced by PAX and RESV promote activation of TRPM2 in the DBTRG cell line. Therefore, we observed synergic interactions of RESV on PAX-induced apoptosis and oxidative stress through stimulation of the TRPM2 channel. These changes contribute to GBM cell death by increasing the influx of Ca^2+^ into the cell through the channel. If bioavailable forms are made available, RESV is likely to be useful in combination with chemotherapeutic agents such as PAX to improve the therapeutic effect of chemotherapy in DBTRG glioblastoma cancer cells.

## Data Availability

The data used to support the findings of this study are available from the corresponding author upon request.

## Abbreviations

[Ca^2+^]_i_: Intracellular free Ca^2+^
ACA: N-(p-Amylcinnamoyl)anthranilic acid
ADPR: ADP-ribose
Ca^2+^: Calcium ion
DHR 123: Dihydrorhodamine-123
GBM: Glioblastoma multiforme
JC1: 5′,6,6′-Tetrachloro-1,1′,3,3′-tetraethylbenzimidazolylcarbocyanine iodide
MTT: 3-[4,5-Dimethyl-2-thiazolyl]-2,5-diphenyl-2-tetrazolium bromide
PAX: Paclitaxel
PGC-*α*: Peroxisome proliferator-activated receptor gamma coactivator-1
RESV: Resveratrol
ROS: Reactive oxygen species
TRP: Transient receptor potential
TRPM2: Transient receptor potential melastatin 2
W.C: Whole cell.
